# Effects of Dietary Copper Deficiency on Colonic Barrier Integrity, Inflammatory Markers, and Gut Microbiota Composition in Mice

**DOI:** 10.3390/nu18111707

**Published:** 2026-05-27

**Authors:** Yaodong Hu, Tianyu Li, Shi Tang, Anqiang Lai, Caiyun Sun, Binlong Chen, Binjian Cai, Li Zhang, Heng Yin

**Affiliations:** 1 College of Animal Science, Xichang University, Xichang 615000, China; mszjhua@163.com (T.L.); tangshi1989103@163.com (S.T.); laq3521@126.com (A.L.); suncaiyun1237@gmail.com (C.S.); binlong2369@163.com (B.C.); cai050806@126.com (B.C.); 2 College of Life Sciences and Agri-Forestry, Southwest University of Science and Technology, Mianyang 621010, China; zl9588623@163.com (L.Z.); yinheng@swust.edu.cn (H.Y.)

**Keywords:** copper deficiency, barrier function, inflammation, gut microbiota

## Abstract

**Introduction:** This study sought to explore the impact of dietary Cu deficiency on colonic health, including assessments of histopathology, barrier function, inflammatory response, and gut microbiota composition. **Methods:** Weaned mice were fed a copper-deficient diet for four weeks, followed by one week of intraperitoneal copper sulfate administration as a proof-of-concept rescue intervention. Colonic pathology was assessed by H&E staining, goblet cell changes by AB-PAS staining, and intestinal barrier integrity by immunofluorescence. Inflammatory cytokine levels were measured by ELISA, while protein and mRNA expression of inflammatory markers were detected by Western blot and qRT-PCR. Gut microbiota composition, diversity, and signature genus abundance were analyzed by 16S sequencing. **Results:** Compared to the control group, CuD mice exhibited histopathological damage in the colon, including mucosal thinning and inflammatory cell infiltration. The number of goblet cells and the expression of mucin MUC2 were significantly reduced, and the expression of tight junction proteins (ZO-1, Occludin) was downregulated, indicating impairment of both the physical and chemical intestinal barriers. Concurrently, Cu deficiency markedly elevated systemic and colonic levels of pro-inflammatory cytokines (TNF-α, IL-1β, and IL-6), and enhanced NF-κB phosphorylation. To explore potential microbial contributions to these colonic alterations, we subsequently analyzed the gut microbiota composition by 16S rRNA sequencing. This analysis revealed that Cu deficiency significantly reduced the α-diversity and species richness of the gut microbiota. This dysbiosis was characterized by a decreased abundance of beneficial bacteria (e.g., Bacteroidota, Muribaculaceae) and an increased abundance of Desulfobacterota, a pro-inflammatory taxon, as well as Akkermansia, a mucin-degrading bacterium with context-dependent effects on gut health. Intraperitoneal administration of copper sulfate (CuD + CuSO_4_) partially reversed the histopathological and inflammatory changes; its effect on the gut microbiota was not assessed. **Conclusions:** Dietary Cu deficiency is associated with colonic injury, and these alterations were accompanied by intestinal barrier disruption, an activated inflammatory response, and gut microbiota dysbiosis. These findings provide experimental evidence highlighting the importance of copper nutrition in maintaining colonic homeostasis, though further mechanistic studies are needed to establish causal relationships.

## 1. Introduction

Copper (Cu) is an essential trace element and a critical cofactor for a variety of key enzymes, such as cytochrome C oxidase, superoxide dismutase, and tyrosinase, and is involved in numerous physiological processes including redox reactions, energy metabolism, neurotransmitter synthesis, and connective tissue formation. It is crucial for maintaining the health of the nervous system, hematopoietic system, and bones [1,2]. Under physiological conditions, the body tightly regulates Cu homeostasis through intestinal absorption, hepatic storage, and biliary excretion [3]. However, long-term dietary Cu deficiency, impaired intestinal absorption (e.g., due to Menkes disease or post-gastrectomy), or specific nutritional conditions (e.g., long-term parenteral nutrition) can induce Cu deficiency, potentially leading to multiple systemic dysfunctions including anemia, osteoporosis, neurodegenerative changes, and immunodeficiency [4].

A tight bidirectional relationship exists between Cu and immune function. Under physiological conditions, adequate Cu supports normal immune responses through mechanisms such as maintaining ceruloplasmin synthesis and antioxidant defense [5]. However, disruption of Cu homeostasis—whether deficiency or excess—can disturb immune balance. In Cu deficiency, the pro-inflammatory interaction between neutrophils and microvascular endothelial cells is enhanced, the expression of cyclooxygenase-2 (COX-2) in the liver is significantly upregulated in an SOD1-independent manner [6], the expression of pro-inflammatory and fibrinogen-related genes is increased [7], and nuclear factor kappa-B (NF-κB) protein levels are elevated [8]. Conversely, Cu excess can also induce gut microbiota dysbiosis and inflammation [9,10]. This bidirectionality suggests that the maintenance of Cu homeostasis, rather than the absolute level of Cu itself, is key to immune regulation. Furthermore, in various inflammatory diseases, serum Cu levels are often compensatorily elevated to several times the normal physiological range [11], further corroborating the close link between Cu and inflammatory regulation.

The intestinal tract serves as the primary site for dietary Cu digestion and absorption, and it is also a key organ for the body to regulate Cu homeostasis and excrete excessive Cu. In addition, Cu plays a complex and crucial role in maintaining intestinal homeostasis, which is essential for the stability of intestinal morphology and structure as well as the microbial community. Excessive Cu exposure can induce intestinal microbiota dysbiosis, characterized by a reduction in probiotic abundance, a decreased Firmicutes/Bacteroidetes (F/B) ratio, and altered populations of bacteria associated with lipid metabolism and intestinal inflammation, ultimately contributing to the development of intestinal inflammation [12]. Dietary supplementation with a high dose of Cu (120 mg/kg) can lead to the accumulation of unabsorbed Cu in the intestinal lumen, which significantly reduces the α-diversity and species richness of the gut microbiota, notably decreasing the abundance of anaerobic commensals such as *Clostridium* [13]. Physiological Cu supplementation, particularly during the weaning period, has been shown to improve intestinal morphology and structure, potentially through promoting the proliferation of crypt epithelial cells and enhancing the self-renewal activity of intestinal stem cells [14].

As a critical segment of the intestine, the colon serves as a key organ for digestion, immune defense, and microbial colonization, with its homeostasis reliant on an intact mucosal barrier, an optimal immune response, and a balanced gut microbiota. Given that the colon is particularly susceptible to dietary influences and microbial dysbiosis, understanding how Cu deficiency specifically affects colonic homeostasis is of considerable importance**.** Evidence suggests that Cu is associated with the occurrence and development of colon diseases such as inflammatory bowel disease, but systematic experimental evidence on how Cu deficiency affects colon morphology and structure, barrier function, and the gut microbiome is still limited. Therefore, this study was designed to establish a Cu-deficient mouse model to assess the impact of Cu deficiency on colonic histopathology, goblet cell function, mucosal barrier integrity, inflammatory responses, and the composition and structure of the intestinal microbiota. The findings aim to elucidate the role of Cu deficiency in colonic homeostasis disruption, providing a foundation for future studies on copper–gut interactions.

## 2. Methods

### 2.1. Animals and Treatments

**Figure 1:**
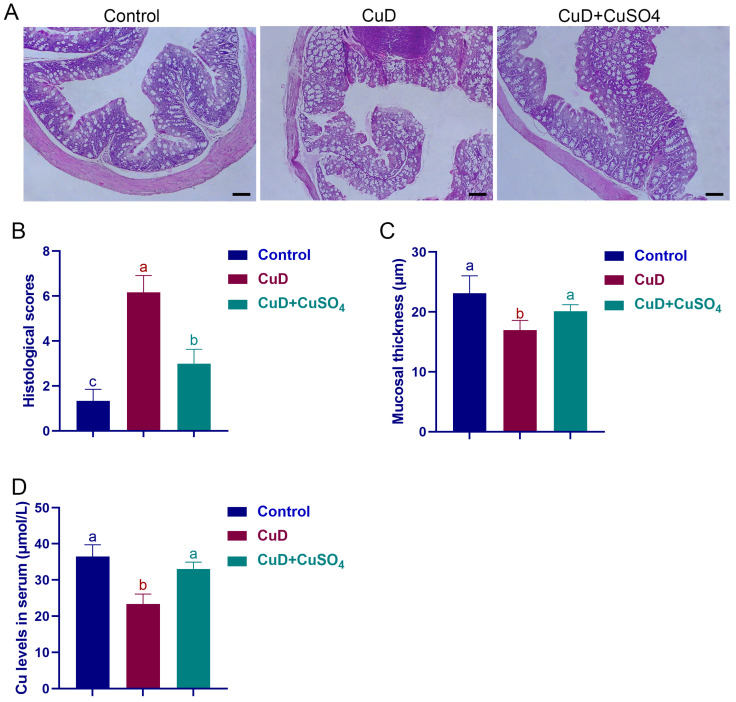
**Effect of Cu deficiency on colonic histology.** Notes: (**A**) H.E. staining (scale bar = 100 µm); (**B**) histological scores; (**C**) Mucosal thickness; (**D**) Serum copper concentration. Data are presented as mean ± SEM (n = 6). Different letters indicate significant differences within a column (*p* < 0.05), while the same letter indicates no significant difference (*p* > 0.05).

Three-week-old healthy male ICR mice (16–18 g) were obtained from Dashuo Biological Technology Company (Chengdu, China). All mice were housed in a specific pathogen-free facility under controlled environmental conditions (temperature: 25 ± 2 °C; 12 h light/dark cycle) with unrestricted access to standard chow and water. Mice were excluded if they had pre-existing abnormalities (e.g., abnormal baseline body weight, congenital defects, or traumatic injury) or experienced non-experimental death during the study. The fifty-four mice were randomly divided into control, Cu-deficient (CuD), and CuD + CuSO_4_ three groups, with 18 mice per group. To ensure experimental reproducibility and statistical robustness, each experimental group included three independent biological replicates (each replicate comprised one cage of six mice). To prevent selection bias, an independent investigator implemented random allocation through a computer-generated randomization process using Microsoft Excel. The sample size was calculated with G*Power software (version 3.1.9.7). Following Cohen’s established criteria for effect magnitudes in ANOVA, a prudent and moderately large effect size (f = 0.4) was applied to secure sufficient statistical power. Under these conditions, with a significance level (α) of 0.05 and 80% power, the analysis determined that a minimum of 22 mice per group would be needed. However, subsequent studies employing the same copper-deficiency protocol have detected significant differences in key outcome measures with as few as 6 mice per group [15], indicating that the actual effect size in this model is considerably larger than the conservative f = 0.4 estimate. Therefore, in keeping with the Reduction tenet of the 3R principles and based on this empirical evidence, the final sample size was set at 18 mice per group. For each experimental assay, 6 mice per group (biological replicates) were used, and all findings are based on this sample size. The animal feeding intervention protocols and copper supplementation dosages and administration routes were designed and implemented in accordance with the methodological system established by Pan et al. [16]. The control group was fed with a standard pellet diet for 5 weeks and injected with saline in the last week. During the final week, the CuD group received daily intraperitoneal injections of saline, while the CuD + CuSO_4_ group received daily intraperitoneal injections of copper sulfate (CuSO_4_, 10 μg/g body weight). The intraperitoneal route was selected to bypass potential intestinal absorption impairments that may persist after prolonged copper deficiency, thereby ensuring controlled systemic copper delivery. Our data confirmed that this one-week regimen restored serum copper to levels comparable to the Control group (Figure 1D). The CuD + CuSO_4_ group was included as a proof-of-concept rescue condition to assess the reversibility of copper-deficiency-induced changes, rather than to mimic dietary copper repletion. After the designated interventions, mice were weighed and then anesthetized with isoflurane. Blood samples were collected from the orbital sinus using sterile 1.5 mL centrifuge tubes. Subsequently, euthanasia was performed by cervical dislocation to minimize suffering, in accordance with the Refinement principle. For tissue allocation within each group of 18 mice, six mice provided colon tissues for H&E staining and immunofluorescence staining, six mice provided colon tissues and colonic contents for quantitative PCR and microbiome analysis, and the remaining six mice provided colon tissues for Western blotting. All procedures were conducted in strict compliance with institutional animal care and use guidelines approved by the Animal Ethics Committee of Southwest University of Science and Technology, Mianyang, China (Approval No.: L2024014). Due to the complexity of the in vivo physiological processes investigated in this study, no suitable non-animal alternatives were available to fully address the research questions, thereby necessitating the use of animal models in compliance with the Replacement principle. The experimental diets were supplied by SPF Biotechnology Co., Ltd. (Beijing, China) and fully met the nutritional standards specified in the AIN-93M maintenance diet. The results of Cu content analysis in the feed are shown in Figure S1.

### 2.2. Histopathological Observation

Colon samples were fixed in 4% paraformaldehyde, then dehydrated through an ethanol series, embedded in paraffin, sliced into 5-μm sections, and stained with hematoxylin-eosin. Blinded histopathological analysis was carried out at 100× magnification using a Nikon DS-Ri1 microscope (Tokyo, Japan), with semi-quantitative ratings of 0 to 3 applied to four criteria: inflammatory cell infiltration, epithelial tissue loss, crypt structural damage, and reduction in mucosal thickness. The total histology injury score, ranging from 0 (no pathology) to 12 (severe damage), was determined by summing the four parameter scores, based on an adapted reference method [17]. For each colon sample, five non-overlapping images were randomly captured at 100× magnification using a light microscope. Mucosal thickness was measured from the base of the crypts to the luminal surface using Image Pro Plus version 6.0 software (Media Cybernetics, Rockville, MD, USA). All measurements were performed by an investigator blinded to the experimental groups, and the mean value of five measurements per sample was used for statistical analysis.

Goblet cells in colonic tissue sections were identified via combined Alcian Blue and periodic acid–Schiff (AB/PAS) histochemistry. Briefly, deparaffinized sections were incubated with 1% Alcian Blue solution (pH 2.5) for 5 min, rinsed thoroughly with distilled water, treated with 1% periodic acid for oxidation, washed again, and then exposed to Schiff’s reagent. After final water rinsing and dehydration, sections were coverslipped and examined under bright-field microscopy. Acid mucins in goblet cells appeared blue, enabling clear identification and assessment. Images were captured and analyzed in a blinded manner with respect to group allocation. From each colon specimen, five representative tissue sections were prepared. For each section, five non-overlapping fields were randomly selected and imaged at 400× magnification. Only goblet cells with well-defined morphology and distinct blue staining (indicative of acidic mucin content) were included in the quantification. Cell counting was performed manually by a blinded observer using standardized criteria, following a protocol adapted from Yin et al. [18].

### 2.3. Serum Cu Concentration

Mice were anesthetized and blood was collected from the retro-orbital plexus. Clotted blood was centrifuged at 3000× *g* for 10–15 min to separate serum, which was then stored at −80 °C until analysis. Serum Cu concentration was determined using a commercially available colorimetric assay kit (E010-1-1, Nanjing Jiancheng Bioengineering Institute, Nanjing, China), following the manufacturer’s protocol.

### 2.4. Immunofluorescent Staining

Mouse colon tissue sections underwent deparaffinization, antigen retrieval in citrate buffer (pH 6.0) via thermal treatment, equilibration to room temperature, and PBS rinsing. Following a 30 min blocking step with 5% goat serum, the sections were incubated at 4 °C overnight with primary antibodies targeting MUC2, ZO-1, CD11b, and occludin. On the following day, the tissue sections underwent three PBS washes and were subsequently exposed to fluorescently labeled secondary antibodies for 30 min at ambient temperature under light-protected conditions. Fluorescent signal was enhanced using a commercial development reagent (10 min, dark), nuclei were stained with DAPI for 10 min in the dark, and the slides were sealed with an antifade mounting medium prior to fluorescence imaging.

For each colon tissue sample, three non-adjacent sections were selected, and five random fields of view (400× magnification) were captured per section. All images were acquired using the same microscope (Olympus, Japan) with identical acquisition settings (exposure time, gain, and fluorescence intensity thresholds) across all samples to ensure comparability. Fluorescence intensity was quantified using ImageJ 1.8.0 software by an investigator blinded to the experimental groups. The mean intensity values from all fields per sample were used for statistical analysis to avoid pseudoreplication.

### 2.5. Cytokine Levels in Serum

Serum samples were processed identically to those described in Section 2.3. IL-1β, IL-6, TNF-α, IL-4, IL-10, and nitric oxide (NO) concentrations were quantified using commercially available ELISA kits (Biosharp, Hefei, China), according to the supplier’s protocol. All assays were performed in technical triplicate, and mean values were calculated for statistical analysis.

### 2.6. Western Blotting Analysis

Colon tissue samples were homogenized in RIPA lysis buffer to extract total protein, and protein concentration was determined using the BCA method (P0010S, Beyotime, Shanghai, China). Equal protein quantities were separated by SDS-PAGE and transferred electrophoretically to nitrocellulose membranes. The membranes were blocked for 1 h at room temperature with 5% non-fat dry milk in TBST, then incubated overnight at 4 °C with primary antibodies specific for NF-κB (1:1000, #8242, CST), phospho-NF-κB (1:1000, #3033, CST), IL-1β (1:1000, A22257, Abclonal, Wuhan, China), IL-6 (1:1000, A0286, Abclonal), TNF-α (1:3000, A28059, Abclonal), and β-actin (1:3000, AC026, Abclonal). Afterwards, the membranes were probed for 1 h with horseradish peroxidase (HRP)-labeled secondary antibodies, and immunoreactive bands were detected using an enhanced chemiluminescence (ECL) kit (P0018A, Beyotime, China) on a ChemiDoc XRS imaging system. Image acquisition and quantification were performed blindly, with the analyst unaware of group assignments, and protein expression levels were normalized to β-actin.

### 2.7. qRT-PCR Analysis

Colon tissues were flash-frozen in liquid nitrogen, homogenized in a pre-chilled mortar and pestle, and stored at −80 °C. Total RNA was extracted from samples using RNAiso Plus (Takara, Shiga, Japan; Cat. No. 9108/9109) according to the supplier’s instructions. Subsequently, 1 µg of purified RNA was reverse-transcribed into cDNA with the PrimeScript RT Reagent Kit (Takara, Japan; Cat. No. RR047A). Quantitative real-time PCR (qRT-PCR) was carried out on a LightCycler^®^ 480 System (Roche, Basel, Switzerland) using SYBR Premix Ex Taq II (DRR820A, Takara, Japan). Primer sequences are provided in Table 1. Target gene expression was normalized to β-actin as an internal reference, and relative mRNA expressions was quantified via the 2^−ΔΔCt^ comparative quantification method.

### 2.8. Determination and Analysis of Gut Microbiota

Colonic luminal contents were collected from mice and immediately frozen at −80 °C. Microbial DNA was extracted using a commercial stool DNA kit. DNA concentration and quality were measured with the Qubit dsDNA HS Assay Kit on a Qubit 4 Fluorometer. The V3–V4 hypervariable region of the bacterial 16S rRNA gene was amplified and subjected to high-throughput sequencing on the Illumina NovaSeq 6000 platform. Bioinformatic analysis was performed on the Novogene Cloud Platform (https://www.novogene.com/eu-en/technology/platforms/, accessed on 10 May 2024). Following read splicing, filtering, and sample demultiplexing, Operational Taxonomic Unit (OTU) clustering was performed. Based on the resulting OTU clusters, taxonomic classification was conducted, and the OTU abundance matrix was subsequently used for downstream analyses. The resulting valid data were subjected to species annotation and abundance analysis to determine the species composition of the samples. Multiple alpha diversity metrics were calculated based on the OTU data, and sequencing depth was assessed through rarefaction analysis. Specifically, alpha diversity metrics (observed species, Chao1 index, Shannon index, and Simpson index) as well as beta diversity based on Bray–Curtis distance were calculated using the R package vegan. Principal coordinate analysis (PCoA) was used to visualize the beta diversity results, and permutational multivariate analysis of variance (PERMANOVA) was adopted to statistically compare the differences in microbiota structure. Taxa with significantly differential abundance were identified by linear discriminant analysis effect size (LEfSe) analysis, with the screening criteria set as an LDA score > 4 and *p* < 0.05.

### 2.9. Statistical Analysis

Data are presented as mean ± standard error of the mean (SEM). For each experimental group, 18 mice were housed in three cages (6 mice per cage). For each assay, a stratified random sampling approach was used: 2 mice were randomly selected from each cage, yielding a total of 6 mice per assay. This ensured representation from all cages and balanced potential cage effects. The experimental unit for all analyses was the individual mouse (n = 6 per group per assay). Differences between group means were assessed using one-way analysis of variance (ANOVA), followed by Tukey’s honestly significant difference (HSD) test for post hoc comparisons. Dunnett’s T3 test was applied when the assumption of homogeneity of variances was violated. All statistical tests were conducted with SPSS 22.0 (IBM Corp., Armonk, NY, USA), and differences were considered statistically significant at *p* < 0.05.

## 3. Results

### 3.1. Effects of Cu Deficiency on Colonic Histology in Mice

Figure 1A–C present the H&E-stained colon sections. Relative to the Control group, mice in the CuD group displayed marked histological abnormalities—such as focal inflammatory cell infiltration and reduced thickness of both the mucosal and muscular layers. In contrast, intraperitoneal CuSO_4_ administration (CuD + CuSO_4_ group) markedly attenuated these pathological changes. Quantitative histopathological assessment confirmed that the CuD group exhibited a significantly higher score compared with the Control group (*p* < 0.05), while the CuD + CuSO_4_ group exhibited a statistically significant decrease in lesion severity compared to the CuD group (Figure 1B). In addition, as shown in Figure 1C, the mucosal thickness in the Cu-deficient group was significantly lower than that in the control group (*p* < 0.05), but there was no statistically significant difference compared with the CuD + CuSO_4_ group (*p* > 0.05).

### 3.2. Effects of Cu Deficiency on the Concentrations of Cu in Serum

Serum Cu concentrations are presented in Figure 1D. A statistically significant reduction in circulating Cu levels was observed in the CuD group relative to the Control group (*p* < 0.05). In contrast, no significant difference in serum Cu was detected between the Control and CuD + CuSO_4_ groups (*p* > 0.05).

### 3.3. Effect of Cu Deficiency on Colonic Goblet Cells in Mice

**Figure 2:**
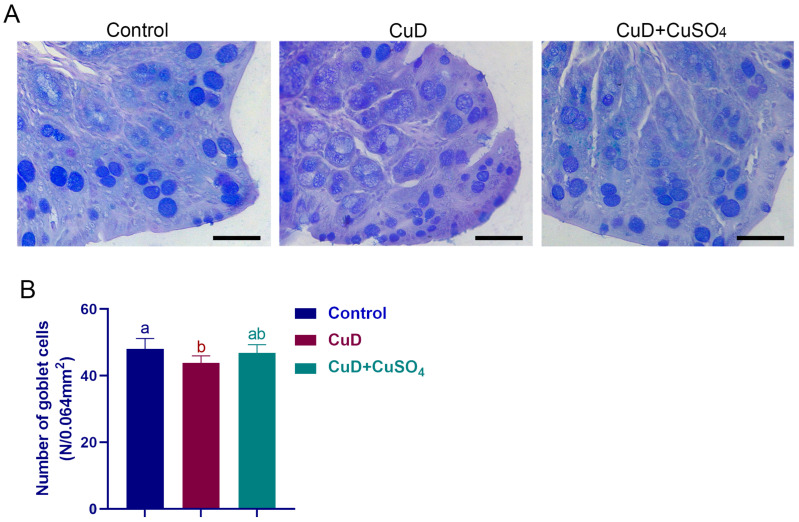
**Effect of Cu deficiency on colonic goblet cells in mice.** Notes: (**A**) Staining of colonic goblet cells (AB/PAS staining, scale bar = 50 µm); (**B**) Changes in the number of goblet cells. Data are presented as mean ± SEM (n = 6). Different letters indicate significant differences within a column (*p* < 0.05), while the same letter indicates no significant difference (*p* > 0.05).

Figure 2A presents the AB–PAS staining results, which specifically highlight acidic and neutral mucins in colonic goblet cells. In the CuD group, the staining of some goblet cells in the intestinal mucosal epithelium was relatively light, indicating a reduction in goblet cell secretions. Quantitative analysis demonstrated a significant reduction in the number of goblet cells in the CuD group compared with the control group (*p* < 0.05) (Figure 2B). In contrast, the CuD + CuSO_4_ group showed no significant difference from the control group (*p* > 0.05).

### 3.4. Effect of Cu Deficiency on Colonic Mucin and Tight Junction Proteins

**Figure 3:**
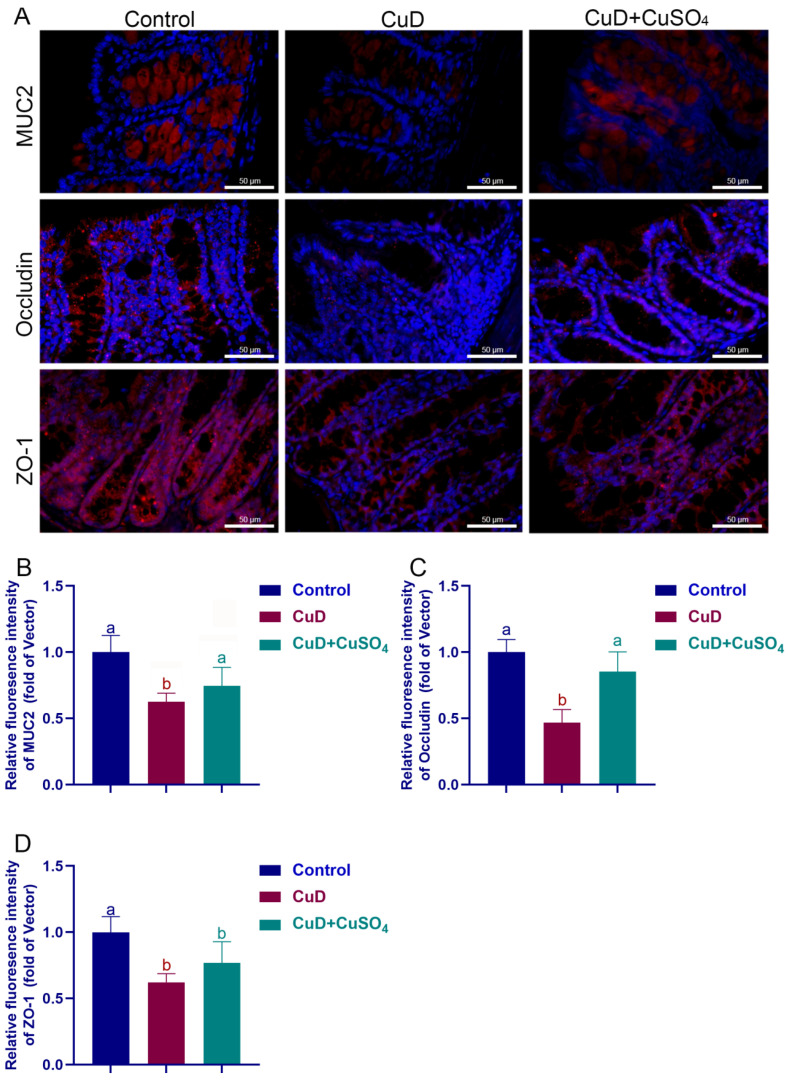
**Effect of Cu deficiency on colonic mucin and tight junction proteins.** Notes: (**A**) Immunofluorescent detection of MUC2, occludin, and ZO-1 (Bar = 50 µm); (**B**–**D**) The fluorescence intensity. Data are presented as mean ± SEM (n = 6). Different letters indicate significant differences within a column (*p* < 0.05), while the same letter indicates no significant difference (*p* > 0.05).

Immunofluorescence analysis revealed a significant reduction in MUC2 expression in colonic tissues of the CuD group compared with the Control group (*p* < 0.05; Figure 3A,B). In contrast, MUC2 expression in the CuD + CuSO_4_ group did not differ significantly from that of the Control group (*p* > 0.05).

Similarly, the fluorescence intensities of ZO-1 and Occludin were markedly lower in the CuD group relative to the Control group (*p* < 0.05; Figure 3A,C,D). However, these reductions were fully rescued in the CuD + CuSO_4_ group, which exhibited ZO-1 and Occludin levels comparable to those in the Control group (*p* > 0.05).

### 3.5. Effect of Cu Deficiency on Inflammation in Mice

**Figure 4:**
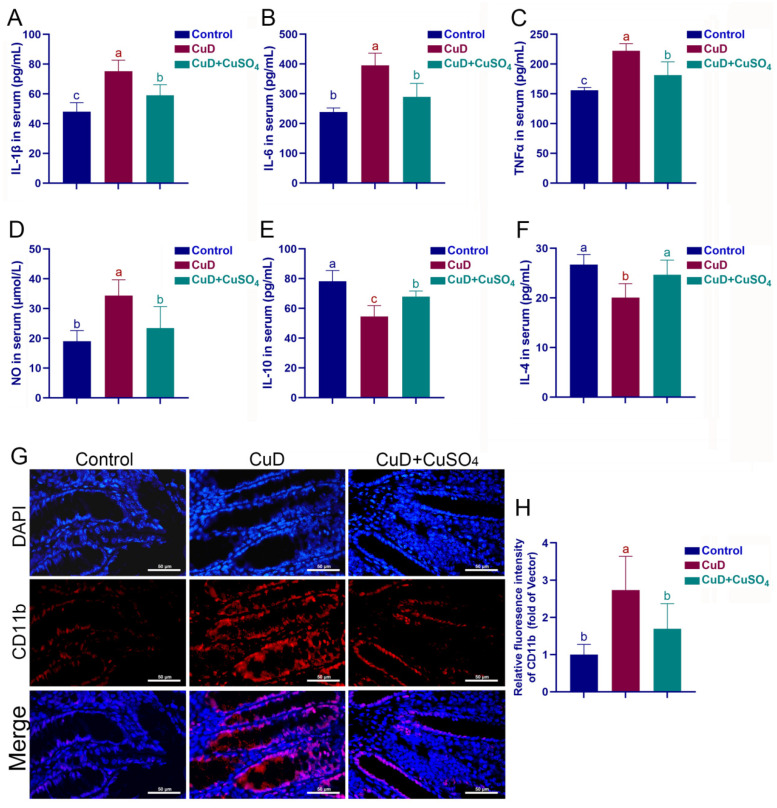
**Effect of Cu deficiency on inflammation in mice.** Notes: (**A**–**F**) Serum concentrations of IL-1β, IL-6, TNF-α, NO, IL-10, and IL-4. (**G**) Fluorescence staining of CD11b, Blue indicates nuclei, red indicates CD11b-positive protein. (**H**) The fluorescence intensity of CD11b. Data are presented as mean ± SEM (n = 6). Different letters indicate significant differences within a column (*p* < 0.05), while the same letter indicates no significant difference (*p* > 0.05).

Serum levels of inflammatory markers were presented in Figure 4A–F. IL-1β, IL-6, TNFα and NO concentrations were significantly elevated in the CuD group relative to the Control group (*p* < 0.05), whereas the concentrations of anti-inflammatory cytokines, including IL-4 and IL-10, were markedly reduced (*p* < 0.05).

Infiltration and accumulation of immune cells in the intestinal mucosa represent hallmark events in intestinal inflammatory responses. To assess this process, we performed immunofluorescent staining to quantify CD11b-positive immune cells in mouse colonic tissues (Figure 4G). Consistent with this, Figure 4H shows a marked increase in CD11b fluorescence signal in the CuD group relative to the Control group (*p* < 0.05).

### 3.6. Effect of Cu Deficiency on Inflammatory Markers in the Colon

**Figure 5:**
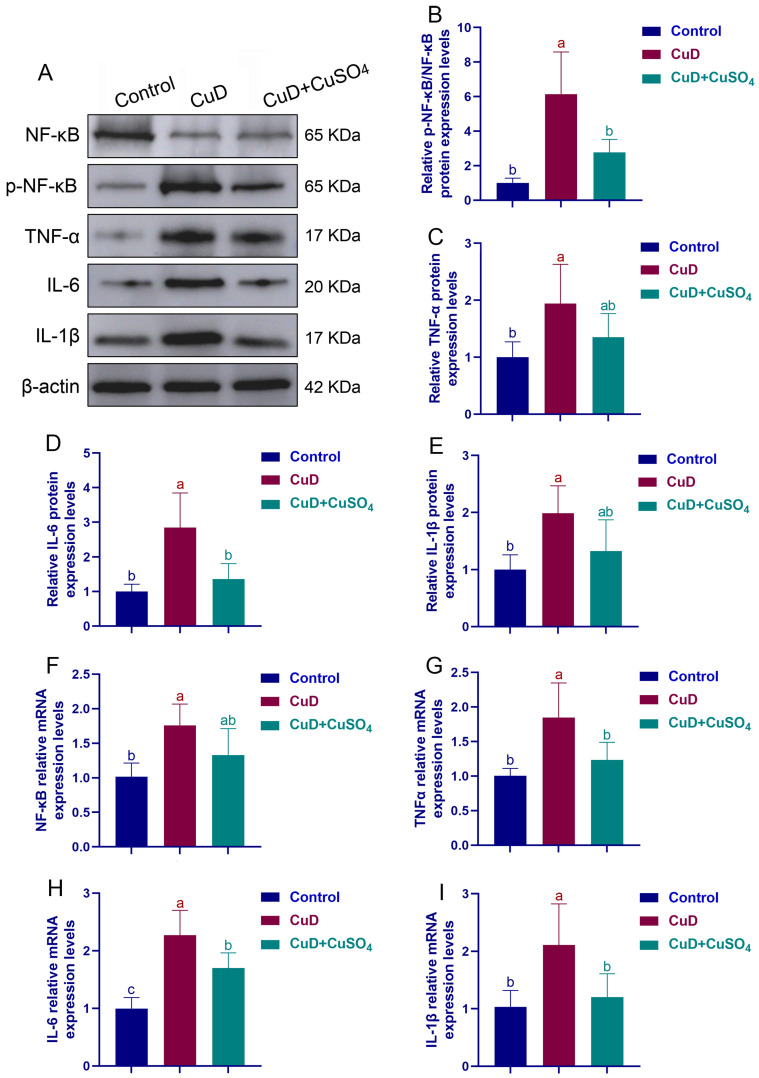
**Effect of Cu deficiency on Inflammatory markers in the colon.** Notes: (**A**) The representative band pictures of inflammatory markers-related molecules; (**B**–**E**) The quantification of *p*-NF-κB, NF-κB, TNF-α, IL-1β, and IL-6 protein expression. (**F**–**I**) The mRNA expression of NF-κB, TNF-α, IL-1β, and IL-6. Data are presented as mean ± SEM (n = 6). Different letters indicate significant differences within a column (*p* < 0.05), while the same letter indicates no significant difference (*p* > 0.05).

As shown in Figure 5A–E, the *p*-NF-κB/NF-κB, TNF-α, IL-1β, and IL-6 protein expressions were significantly elevated in the CuD group compared to the control group (*p* < 0.05). However, no significant difference was observed in the expression levels of these proteins between the CuD + CuSO_4_ group and the control group (*p* > 0.05).

Consistent with this, mRNA level analysis (Figure 5F–I) revealed significantly elevated mRNA expression levels of NF-κB, TNF-α, IL-1β, and IL-6 in the CuD group relative to the control group (*p* < 0.05).

### 3.7. Effect of Cu Deficiency on the Composition of the Gut Microbiota in Mice

**Figure 6:**
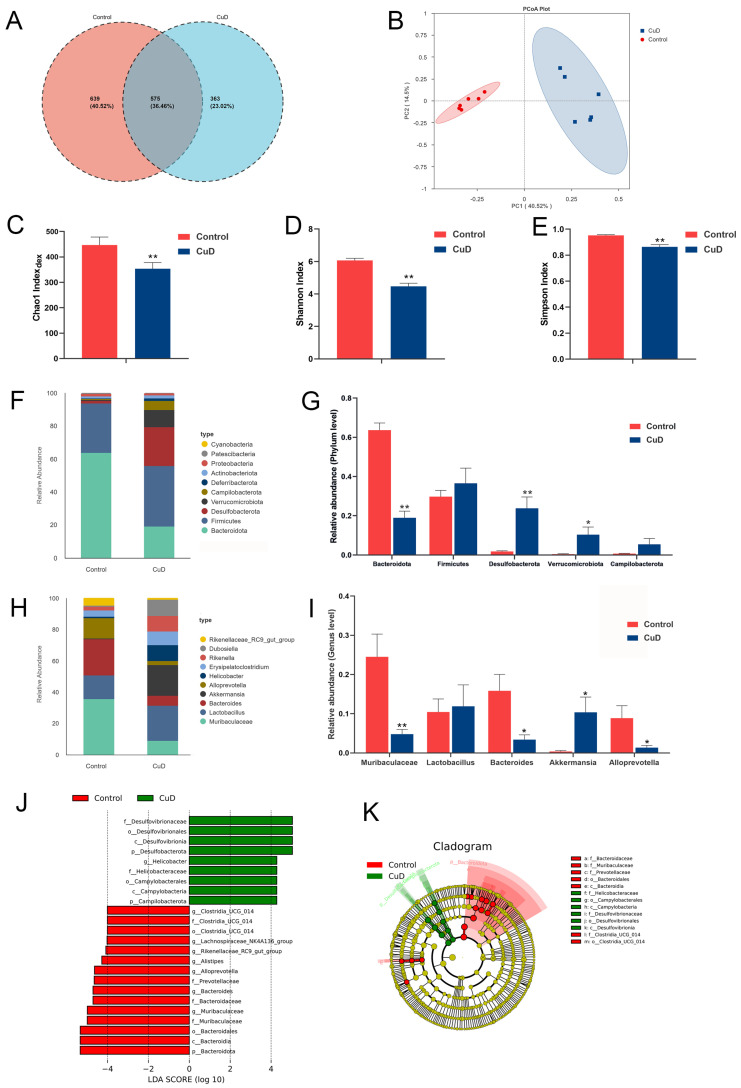
**Effect of Cu deficiency on the composition of the gut microbiota.** Notes: (**A**) OTUs; (**B**) PCoA; (**C**) Chao1 index; (**D**) Shannon index; (**E**) Simpson index; (**F**,**G**) Phylum-level profiling of microbial community structure; (**H**,**I**) Genus-level profiling of microbial community structure; (**J**) LDA score distribution (log10, LDA > 4.0) showing differentially abundant taxa between Control and CuD groups. (**K**) Cladogram showing the phylogenetic distribution of differentially abundant taxa. Data are expressed as the mean ± SEM (n = 6). * *p* < 0.05, ** *p* < 0.01.

As shown in Figure 6A, the Control group displayed 1214 distinct OTUs, whereas the CuD group presented 938, and 575 OTUs were common to all groups. PCoA utilizing weighted UniFrac distances revealed a clear distinction in microbial community profiles between the Control and CuD groups (Figure 6B), pointing to notable compositional shifts. Key alpha-diversity measures, namely the Chao1, Shannon, and Simpson indices, were markedly lower in the CuD group than in the Control group (*p* < 0.01; Figure 6C–E), together indicating that Cu deficiency in the diet contributed to reduced gut microbial richness and diversity in mice.

At the phylum level (Figure 6F,G), the relative abundance of Bacteroidetes was significantly lower in the CuD group than in the control group (*p* < 0.01), whereas those of Desulfobacterota and Verrucomicrobiota were markedly higher (*p* < 0.01 or 0.05). At the genus level (Figure 6H,I), the CuD group exhibited significantly reduced relative abundances of *Muribaculaceae*, *Bacteroides*, and *Alloprevotella* (*p* < 0.01 or 0.05), along with a significant increase in *Akkermansia* relative to the control group (*p* < 0.05). LEfSe analysis further identified taxonomic features that were differentially enriched across the experimental groups (Figure 6J,K). In the CuD group, marked enrichment was observed in specific taxa, including the f_*Desulfovibrionaceae*, p_Desulfobacterota, g_*Helicobacter*, f_*Helicobacteraceae*, o_*Campylobacterales*, c_*Campylobacteria*, and p_Campilobacterota. Conversely, the Control group showed significant enrichment of members within the p_Bacteroidota, c_*Bacteroidia*, o_*Bacteroidales*, f_*Muribaculaceae*, g_*Muribaculaceae*, f_*Bacteroidaceae*, g_*Bacteroides*, f_*Prevotellaceae*, and g_Alloprevotella.

## 4. Discussion

A bidirectional physiological relationship exists between Cu and the gut. The intestine serves not only as the primary site for dietary Cu absorption but also as a key regulator of systemic Cu homeostasis. Conversely, Cu levels and speciation directly influence intestinal structure and function. By establishing a mouse model of dietary Cu deficiency, this study systematically examined the resulting multifaceted detrimental effects on the colon and elucidated the underlying mechanisms.

Histomorphological assessment showed that Cu deficiency was associated with impaired colonic development and pathological changes, most notably a significant decrease in mucosal thickness accompanied by inflammatory cell infiltration. Goblet cells, mucus-secreting epithelial cells abundant in the colon, produce a protective mucus layer that shields the epithelium from gastric acid, bile, proteolytic enzymes, and pathogens, thereby maintaining intestinal barrier integrity. A reduced number of goblet cells reflects mucosal atrophy and weakened mucin (e.g., MUC2) synthesis, thereby promoting intestinal barrier hyperpermeability and local immune dysregulation [18]. The significant reduction in both goblet cell number and AB-PAS staining intensity in the CuD group suggests that copper deficiency may impair goblet cell differentiation or secretory function. Given that goblet cell maturation and mucin synthesis are energy-intensive processes, copper depletion—via its effects on mitochondrial respiration—may limit the metabolic capacity of these cells to produce and secrete mucins. Additionally, the observed reduction could partially reflect epithelial atrophy, as indicated by the decreased mucosal thickness in the CuD group**.** To further explore the relationship between Cu deficiency and colonic barrier integrity, this study detected the expression of mucin MUC2 and tight junction proteins. MUC2 is a key structural component of the intestinal mucus layer, and its reduced expression indicates that the integrity of the mucus barrier may be compromised [19]. Tight junctions, which are primarily composed of proteins including ZO-1 and Occludin, constitute essential structural elements of the intestinal epithelial barrier by regulating paracellular permeability and maintaining mucosal integrity [20]. The downregulation of MUC2, Occludin, and ZO-1 in the CuD group suggests that copper deficiency may compromise the intestinal barrier at multiple levels. MUC2 reduction likely reflects impaired goblet cell secretory function, while the loss of tight junction proteins may result from either direct effects of copper depletion on epithelial cell homeostasis or secondary consequences of heightened local inflammation, given that pro-inflammatory cytokines such as TNF-α are known to disrupt tight junction assembly. The combined impairment of the chemical (mucus) and physical (tight junction) barriers may create a self-reinforcing cycle, where a weakened barrier permits greater luminal antigen translocation, further fueling inflammation. Interestingly, both copper deficiency and excess appear to compromise colonic integrity. Early studies found that long-term low dietary Cu significantly increased the incidence of colon tumors in rats and caused colonic abnormalities [20]. Liao et al. [21] reported that excessive Cu intake impairs the colonic barrier function in pigs, as evidenced by reduced expression of tight junction proteins (ZO-1, Occludin, Claudin-1, and JAM-1), Muc2, and mucus secretion-related genes. Taken together, disruption of Cu homeostasis, whether through deficiency or excess, may be associated with compromised colonic barrier integrity. Furthermore, this research provides experimental evidence supporting a potential role of Cu deficiency in relation to the development and maintenance of colonic barrier integrity.

The impairment of intestinal barrier function facilitates the translocation of luminal microbiota and antigens, which activate innate immunity and contribute to the development of inflammatory bowel disease [22]. To investigate the impact of Cu deficiency on colonic inflammation, this study systematically assessed key inflammatory markers. The elevated systemic levels of pro-inflammatory cytokines (IL-1β, IL-6, TNF-α) and NO, coupled with suppressed anti-inflammatory cytokines (IL-4, IL-10), suggest that copper deficiency induces a systemic inflammatory state. The concurrent upregulation of CD11b in colonic tissue indicates that this inflammatory response is, at least in part, localized to the intestine. The activation of NF-κB phosphorylation observed in the CuD colon provides a plausible mechanistic link: copper deficiency may lower the threshold for NF-κB activation, as Cu-dependent antioxidant enzymes normally restrain redox-sensitive inflammatory signaling. The resulting pro-inflammatory milieu could then contribute to the barrier disruption discussed above. Moreover, the significant increase in both mRNA and protein expression of IL-1β and IL-6 in colon tissue further evidenced the activation of intestinal inflammation. Collectively, our results indicate that Cu deficiency can induce systemic inflammation accompanied by the occurrence of colonic inflammatory responses, while the causal relationship between the two remains to be further explored. NF-κB serves as a central regulator of inflammatory responses and is intricately involved in key pathological processes, including intestinal barrier impairment and oxidative stress [18,23]. This study confirmed that inflammatory markers (NF-κB and TNF-α) were significantly elevated in Cu-deficient colon tissues, suggesting that Cu deficiency is accompanied by intestinal inflammation. Currently, studies specifically addressing the impact of Cu deficiency on colonic inflammation remain scarce, with the existing literature predominantly centered on the effects of Cu overload or therapeutic supplementation. Zhang et al. [13] reported that feeding high doses of Cu (120 and 240 mg/kg feed) could significantly increase the concentration of pro-inflammatory factors in rat serum, accompanied by disordered fecal microbiota structure, and indirectly promote intestinal inflammation. Similarly, Liao et al. [21] found that excessive Cu leads to reduced secretion of secretory immunoglobulins A (SIgA) and G (SIgG) in the jejunum and colon, along with upregulated expression of inflammatory factors such as IL-1β and TNF-α. Lin et al. [24] found that long-term Cu exposure significantly increased the mRNA levels of TLR3, TLR7, TLR8, NF-κB, I-κB, TNF-α and IL-8 in the gut of marsh eels, triggering intestinal biological damage and inflammatory responses. Notably, Fu et al. [25] showed that supplementation with Cu ion-Lut nanocomplexes could relieve colitis symptoms by inhibiting NF-κB expression and exerting anti-inflammatory and intestinal barrier protective effects. However, the cross-sectional nature of this study precludes determining the temporal sequence of these events. Reduced tight junction protein expression and mucin depletion may facilitate luminal antigen translocation, thereby triggering inflammatory responses [26]. Conversely, inflammation itself can directly disrupt tight junction integrity and goblet cell function through pro-inflammatory cytokines such as TNF-α and IL-1β [27,28]. It is therefore plausible that barrier dysfunction and inflammation operate as a self-amplifying feedback loop under Cu-deficient conditions, rather than following a simple unidirectional causality. Future time-course studies are needed to dissect the initiating events in this process.

Gut microbiota homeostasis is essential for intestinal health, and its disruption is recognized as a key driver of intestinal inflammation [29,30]. To determine whether Cu deficiency contributes to colonic pathological changes through modulation of the gut microbiota, we further examined structural alterations in the intestinal microbial community. The reduced α-diversity and altered community structure in the CuD group may reflect a selective pressure imposed by copper deficiency on the gut microbial ecosystem. Copper serves as an essential cofactor for numerous bacterial enzymes involved in energy metabolism and antioxidant defense [31], and its scarcity may disproportionately affect species with higher copper requirements, thereby reducing community richness. At the phylum level, the marked decrease in Bacteroidetes—a dominant phylum in the healthy gut—and the concomitant expansion of Desulfobacterota suggest a shift toward a pro-inflammatory community configuration. This compositional shift could be driven by both direct effects of copper limitation on bacterial growth and indirect effects mediated by the altered host environment, including a compromised mucus barrier and heightened oxidative stress in the intestinal lumen [32,33]. Most studies report that intestinal inflammation is associated with a marked reduction in colonic Bacteroidetes abundance, and this reduction is negatively correlated with markers of inflammation [34]. In addition, Desulfobacterota can release lipopolysaccharide (LPS) into the gut, which has been associated with promoting inflammatory responses and disrupting intestinal energy metabolism in other studies [35]. Verrucomicrobiota, primarily residing in the inner mucosal layer of the intestine, contribute to energy and nutrient supply by degrading polysaccharides such as mucopolysaccharides and cellulose [36]. Notably, Ren et al. [37] observed a marked increase in Verrucomicrobiota abundance in the colon of mice with DSS-induced colitis. At the genus level, Cu deficiency led to a significant reduction in the relative abundance of *Muribaculaceae*, *Bacteroides*, and *Alloprevotella*, whereas the abundance of *Akkermansia* was markedly increased. *Muribaculaceae* and *Bacteroides*, both members of the *Bacteroidales* order, produce short-chain fatty acids that supply energy to intestinal epithelial cells, and their increased abundance is significantly correlated with enhanced intestinal barrier integrity and reduced inflammation [38,39]. *Alloprevotella* is a gut commensal bacterium that metabolizes dietary proteins and carbohydrates, with butyrate as its primary metabolite, which exerts inhibitory effects on enteric pathogens and plays a critical role in sustaining intestinal homeostasis in the host [40,41]. While *Akkermansia muciniphila* is widely recognized as a beneficial symbiont that promotes gut barrier integrity and metabolic health under normal conditions [42], its role is highly context-dependent. *Akkermansia* is a mucin-degrading specialist; under conditions of compromised mucosal barrier function—such as the reduced MUC2 expression and goblet cell depletion observed in our CuD mice—excessive *Akkermansia* proliferation could hypothetically exacerbate mucus layer thinning by accelerating mucin degradation, potentially further weakening the barrier, although this remains to be directly tested. This is consistent with reports that *Akkermansia* expansion has been observed in certain pathological states where its overgrowth is associated with, rather than protective against, barrier dysfunction [43]. Copper deficiency resulted in a marked reduction in key anti-inflammatory commensals and an increase in pro-inflammatory taxa, which is generally consistent with the findings of Klevay et al. [44], suggesting that copper deficiency is associated with reshaping of the gut microbiota and with colonic inflammation. Causative relationships between specific taxa and inflammatory outcomes cannot be established from our correlational data.

## 5. Conclusions

In summary, this study demonstrates that dietary copper deficiency in mice is associated with impaired colonic barrier integrity, enhanced inflammatory responses, and gut microbiota dysbiosis. Intraperitoneal copper sulfate administration significantly attenuated the histopathological and inflammatory alterations, though its effect on the gut microbiota was not assessed. These findings underscore the importance of adequate copper status for colonic homeostasis. However, the observational nature of this study precludes establishing causal relationships among barrier disruption, inflammation, and microbial dysbiosis. Given the severe deficiency model and single-endpoint analysis employed, direct extrapolation to human physiology should be made with caution. Additionally, because mice were co-housed, gut microbiota profiles from individuals within the same cage are not fully independent. This potential cage effect should be considered when interpreting the microbiome data, and we refrain from overstating the independence of samples. Marginal copper deficiency occurs in specific populations—including individuals with malabsorption syndromes, those on long-term parenteral nutrition, and the elderly with poor dietary intake—yet its impact on intestinal health remains largely unexplored. Future studies should employ time-course and pair-feeding designs to establish temporal and causal relationships among barrier disruption, inflammation, and microbial dysbiosis, and to determine whether these findings extend to at-risk human populations.

## Figures and Tables

**Table 1:** The primer sequences utilized in this study.

Gene	Forward (5′ → 3′)	Reverse (5′ → 3′)	Product Size	Tm (°C)
*NF-κB*	GGGCATGCGTTTCCGTTACA	ATGTGGATGAGGCCGGTGAG	121 bp	59.8
*TNF-α*	CGTCGTAGCAAACCACCAAG	TTGAAGAGAACCTGGGAGTAGACA	95 bp	62
*IL-6*	ACAGAAGGAGTGGCTAAGGA	AGGCATAACGCACTAGGTTT	95 bp	60
*IL-1β*	TCGGCAAAGAAATCAAGATGGC	GTGCAAGTCTCATGAAGTGAGC	132 bp	61
*β-Actin*	GGCTGTATTCCCCTCCATCG	CCAGTTGGTAACAATGCCATGT	117 bp	60.9

## Data Availability

The data supporting the findings of this study are available from the corresponding author upon reasonable request.

## Supplementary Materials

The following supporting information can be downloaded at: https://www.mdpi.com/article/10.3390/nu18111707/s1, Figure S1: Copper concentration in the control and copper-deficient diets. ** denotes a statistically significant difference (*p* < 0.01).
