# Supplementary material for: Effects of Dietary Copper Deficiency on Colonic Barrier Integrity, Inflammatory Markers, and Gut Microbiota Composition in Mice
Source: Nutrients. 2026 May 27;18(11):1707. doi: 10.3390/nu18111707 (PMC13258561; doi:10.3390/nu18111707)
Supplement: Supplementary file 1 [file nutrients-18-01707-s001.zip › nutrients-4309716-supplementary.pdf]

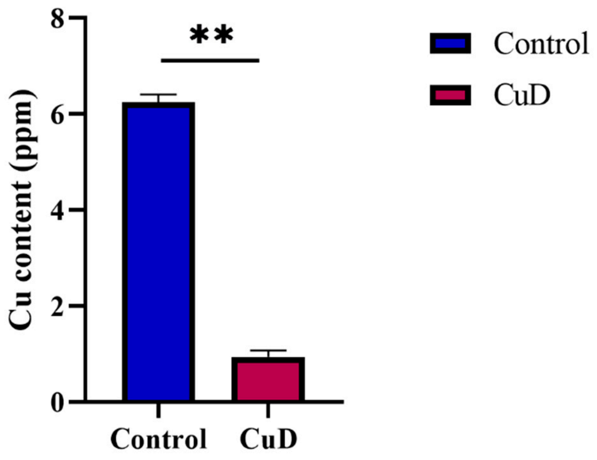

Figure S1 Copper concentration in the control and copper-deficient diets. ** denotes a statistically significant difference ($p < 0.01$).
